# Robust Machine Learning Framework for Modeling the Compressive Strength of SFRC: Database Compilation, Predictive Analysis, and Empirical Verification

**DOI:** 10.3390/ma16227178

**Published:** 2023-11-15

**Authors:** Yassir M. Abbas, Mohammad Iqbal Khan

**Affiliations:** Department of Civil Engineering, College of Engineering, King Saud University, Riyadh 800-11421, Saudi Arabia; yabbas@ksu.edu.sa

**Keywords:** machine learning, steel-fiber-reinforced concrete (SFRC), feature importance, partial dependence plots, prediction model, graphical user interface

## Abstract

In recent years, the field of construction engineering has experienced a significant paradigm shift, embracing the integration of machine learning (ML) methodologies, with a particular emphasis on forecasting the characteristics of steel-fiber-reinforced concrete (SFRC). Despite the theoretical sophistication of existing models, persistent challenges remain—their opacity, lack of transparency, and real-world relevance for practitioners. To address this gap and advance our current understanding, this study employs the extra gradient (XG) boosting algorithm, crafting a comprehensive approach. Grounded in a meticulously curated database drawn from 43 seminal publications, encompassing 420 distinct records, this research focuses predominantly on three primary fiber types: crimped, hooked, and mil-cut. Complemented by hands-on experimentation involving 20 diverse SFRC mixtures, this empirical campaign is further illuminated through the strategic use of partial dependence plots (PDPs), revealing intricate relationships between input parameters and consequent compressive strength. A pivotal revelation of this research lies in the identification of optimal SFRC formulations, offering tangible insights for real-world applications. The developed ML model stands out not only for its sophistication but also its tangible accuracy, evidenced by exemplary performance against independent datasets, boasting a commendable mean target-prediction ratio of 99%. To bridge the theory–practice gap, we introduce a user-friendly digital interface, thoroughly designed to guide professionals in optimizing and accurately predicting the compressive strength of SFRC. This research thus contributes to the construction and civil engineering sectors by enhancing predictive capabilities and refining mix designs, fostering innovation, and addressing the evolving needs of the industry.

## 1. Introduction

Today, the world consumes 14 billion cubic meters of concrete annually, equating to approximately 4.4 tons per individual [1]. Despite its popularity, concrete has some inherent difficulties that limit its use under harsh weather conditions and in modern architectural designs. A major drawback of concrete is its relatively low tensile strength. Reinforced concrete (RC) structures are traditionally designed without considering this property, constituting approximately 6 to 12% of the normal concrete’s compressive strength (CS) [2,3,4]. Using discrete, randomly distributed, and discontinuous steel fibers in concrete reinforces the mix to mitigate problems [5,6,7]. This type of composite material is known as steel-fiber-reinforced concrete (SFRC). Consequently, RC members can be enhanced in crack resistance by using this easy-to-manufacture and highly effective technology [8].

Concrete stands out as the foremost building material employed extensively across the global construction industry, thanks to its unparalleled attributes of durability, strength, and sustainability [9,10,11]. In this framework, steel-fiber-reinforced concrete (SFRC), enhanced with short discrete fibers as mass reinforcement, emerges as an exceptionally effective cement-based composite capable of substantially alleviating the inherent brittleness found in plain concrete [12]. The incorporation of fibers into concrete represents a pivotal enhancement, notably augmenting its strength, toughness, crack resistance, and tension performance. This transformative impact arises from the adept intervention of randomly distributed fibers, skillfully restraining the unstable propagation of cracks, operating seamlessly at both the micro and macro levels [13]. Remarkably, SFRC exhibits a phenomenon known as strain-softening behavior, persisting even after the emergence of macro-cracks—a testament to the significant crack control offered by these fibers [14]. The introduction of steel-fiber reinforcement into concrete goes a step further, markedly elevating its ductility and toughness. The observed result can be chiefly ascribed to the incorporation of additional fracture mechanisms and the energy invested in overcoming the interlocking and adhesive forces existing between the fibers and the cementitious matrix [15]. To evaluate the improvement in ultimate properties, it is imperative to conduct mechanical tests, and the determination can only be ascertained upon the completion of the loading phase. Recent developments have underscored the utility of acoustic emission as a means to evaluate the performance of SFRC beams [16]. Furthermore, it is noteworthy that the formation of macro-cracks has the potential to induce the corrosion of steel reinforcement. Therefore, the reduction in crack width, achieved through the presence of fibers, assumes paramount importance in enhancing the durability of reinforced concrete (RC) structural elements [17]. Furthermore, compelling evidence suggests that the inclusion of steel fibers may reduce the need for conventional steel shear reinforcement. This conclusion is drawn from a comprehensive array of tests and analyses conducted by the authors and other esteemed researchers in the field [18,19,20].

SFRC was patented by Bernard in 1874 for strengthening concrete in tension, using steel splinters to achieve this purpose [21]. In the ensuing years, this exploration has led to many studies being conducted on its microstructure [22,23], flowability, and SFRC’s behavior under tension [24,25], durability [26,27,28], and strength under extreme and cyclic loadings [28,29,30]. In recent years, several new types of fibers have been proposed as reinforcements for SFRC, along with the use of magnetic fields to align the steel fibers during casting [30,31], and micro-scale numerical analyses have been published to illustrate the fundamental failure mechanism of SFRC under varying external loads [32,33]. According to these studies, SFRC has substantially different strength and elasticity properties than traditional concrete. For the post-cracking response, the SFRC is positively influenced by the high tensile resistance and elasticity that results in a crack-bridging mechanism. Hence, this composite material has excellent behavior under tension and shear loadings. A large extent of mechanical property variability is, however, a result of material heterogeneity. A steel-fiber reinforcement can prevent macrocrack propagation in concrete, but the resulting SFRC may be less flowable than its conventional equivalent. The quality reduction is likely caused by the interference between aggregates and fibers [33].

A significant consideration in civil engineering applications revolves around the urgent requirement for vigilance and the implementation of cutting-edge state-of-health identification techniques within current infrastructural frameworks [34]. An effective solution to this challenge involves continuous, real-time surveillance conducted in-situ to detect potential structural issues, including damage from cracking or yielding of steel reinforcements, and subsequently assess their severity levels [35]. The avant-garde technology of structural health monitoring (SHM), utilizing intelligent materials and systems, has become instrumental in assessing the internal state of reinforced concrete (RC) structures under both normal operational conditions and during critical loads. Notably, piezoelectric lead zirconate titanate (PZT) transducers have garnered widespread acclaim in electro-mechanical-admittance-based (EMA-based) health monitoring, primarily due to their favorable attributes [36,37]. Recent studies have showcased the successful deployment of the EMA technique, incorporating small-sized PZT transducers that are either surface-bonded or embedded, to identify damage in RC structural components [34,38]. Incorporating piezoelectric materials into SHM techniques presents a multitude of advantages, encompassing a high-frequency response, structural simplicity, cost-effectiveness, and the capability to generate an electrical signal through the application of mechanical force. However, certain limitations and uncertainties come to the forefront, particularly when applied to cracked, damaged, or non-homogeneous construction materials like RC. Identifying the severity and location of structural damages can prove challenging, as low-level damage occurring near the piezoelectric transducer’s placement can yield similar results to more extensive damage situated farther away [34].

A design-oriented formulation for the key mechanical properties of SFRC is necessary for the calculation of their key performance characteristics to facilitate their successful implementation in practical applications. However, the existing models tend to be either insufficiently accurate or lack physical relevance. In the literature, there have been several studies [39,40,41] proposing linear empirical formulas for predicting SFRC mechanical properties. There is a general consensus that fiber dosage and water-binder ratio play the greatest role in determining SFRC behavior. A composite material mixing theory indicates that SFRC properties strongly correlate with matrix and steel-fiber elastic properties. This theoretical background has been used as a foundation for the development of various empirical models [42]. In spite of the fact that some of the currently available empirical models are capable of accurately predicting the results of a small number of tests [7,43], the development and implementation of comprehensive and robust models are still in the early stages of development.

Machine learning (ML) and ML interpretability algorithms find extensive application in various aspects of structural engineering. These applications encompass a wide range of sectors within the realm of structural engineering, spanning structural analysis, design, monitoring structural health, detecting damage, evaluating fire resistance in structures, assessing the resistance of structural elements under various loads, and examining the mechanical properties as well as the mix design of concrete [44]. As an illustration of the capabilities of these techniques, Zheng et al. [45] successfully employed a YOLO-v5 model to detect surface cracks on wind turbines. Similarly, Cardellicchio et al. [46] leveraged ML to facilitate the recognition and interpretation of defects for the purpose of risk management in heritage bridge preservation.

The use of machine learning (ML) to model SFRC’s mechanical properties has become increasingly popular over the last few years. In this context, the back-propagation artificial neural network (ANN) approach has been utilized to calculate SFRC’s CS by Açikgenç et al. [43]. Their study used the aggregate size at maximum fiber dosage, length, and size, as well as fiber length and size, as input variables. In a similar manner, Awolusi et al. [47] used an ANN method to predict the flowability, CS, and splitting strength of SFRC. In addition, Karahan [48] used multiple nonlinear regression techniques in conjunction with ANN to predict the long-term strength of SFRC containing varying levels of fly ash. While these closed-source databases may be able to provide accurate estimates of SFRC mechanical properties, their limitations remain a concern. There have been criticisms of these models for their deficiencies, which include a lack of a physical mechanism illustration and a lack of an application tool. Recently, Pakzad et al. [7] have employed various data-driven machine-learning algorithms to predict the CS of SFRC. Sensitivity and parametric analyses were performed to demonstrate the capabilities of ML algorithms in their study.

In this study, we present solutions to the above-mentioned by focusing on the CS of SFRC-containing mono-fibrous (crimped, hooked, and mil-cut) systems. The research has been completed in several phases to achieve the goal. First, a comprehensive database of 420 records collected from 43 published studies was compiled and refined to establish a representative sample population. The second component of this research involves constructing and evaluating a numerical model that uses a state-of-the-art ML technique (XG Boost). A third aspect of the study involved narrowly focused experiments designed to verify the developed model using 30 SFRC mixes. Fourth, the model parameters were ranked according to their importance, and partial dependence plots were constructed to visualize their relationships. An intuitive graphical user interface was developed to enhance the model’s applicability.

ML has seen increased application in modeling SFRC properties. However, many current ML models for this purpose are “black boxes” that lack transparency and physical relevance, and also present implementation challenges. Moreover, globally, there is significant consumption of concrete, which, despite its popularity, has inherent limitations, including its relatively low tensile strength. The necessity to improve the transparency, relevance, and implement-ability of ML models for SFRC properties, combined with the global demand for more durable and versatile concrete solutions, drives this research. Existing models for predicting and optimizing SFRC properties using ML lack transparency, do not have a clear physical basis and are challenging to implement in practical scenarios. This study introduces an innovative approach that utilizes the extreme gradient (XG) boosting algorithm for predicting and optimizing the CS of SFRC. The approach is grounded in a comprehensive database, which is a compilation from 43 publications, and is validated through extensive experimental studies. The ML model developed in this research not only promises superior predictive capabilities against independent experimental data but also introduces a user-friendly interface, paving the way for more accessible and efficient predictions and optimizations in the realm of SFRC. This study represents a transformative effort within the construction industry landscape. Although ML techniques have gained prominence in predicting material properties, a significant challenge endures—the lack of transparency and practical relevance in existing models. To address this challenge and enhance real-world applicability, this research adopts a meticulous approach, leveraging the XG boosting algorithm. Furthermore, the investigation gains clarity through the application of partial dependence plots (PDPs), which reveal intricate connections between input parameters and the CS of SFRC. Of utmost importance, the current study uncovers optimal SFRC formulations, offering practical insights into the most effective combinations of fibers for real-world applications.

## 2. Methodological Approach

### 2.1. Background

The extreme gradient boosting (XG Boost) algorithm is a collective ML method that integrates a gradient boosting algorithm with decision trees to convert training data into a regression model suitable for classifying new data [49]. Due to Friedman et al.’s [50] introduction of the gradient boosting methodology, Chen et al. [51] developed an algorithm designed to enhance the performance of the gradient boosting methodology. In comparison to gradient boosting, the XG Boost algorithm can be distinguished from gradient boosting with several advantages, such as efficient tree partitioning, shorter nodes, randomization with Newton–Raphson boosting, and multi-objective optimization [52]. In recent years, it has become a very popular programming language because of its inclusion in Python and its use in several Kaggle [53] competitions. The model has demonstrated excellent predictive capabilities in identifying a person’s medical condition [54], forecasting the effects of the COVID-19 pandemic [55], and predicting a company’s probability of bankruptcy [56].

### 2.2. XG Boost Algorithm Development

**Figure 1:**
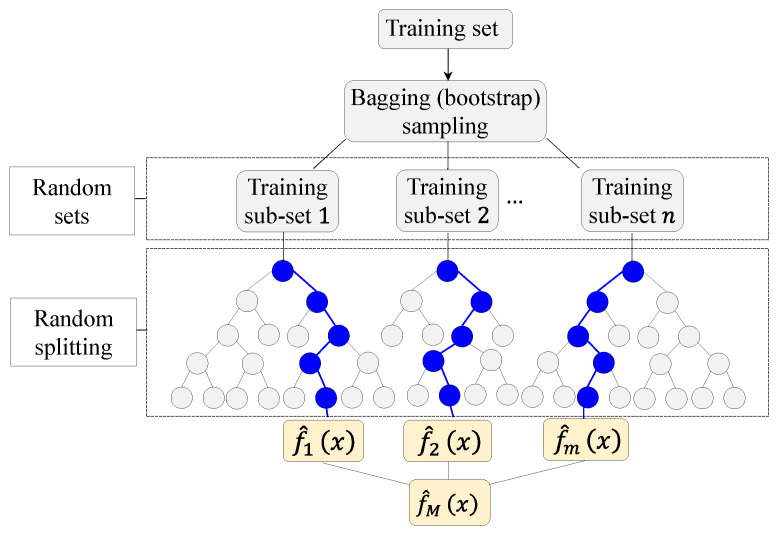
XG Boost methodology.

In Figure 1, a conceptual diagram illustrates the steps involved in the preparation of an XG Boost algorithm, while the following equations provide a detailed formulation of the algorithm. It is noteworthy that Python (version 3.12.0) [57] was used for coding.

In this study, the database of the SFRC involves N variables with $x\in R^{M\times N}$ and the model’s output, $y\in R^{M\times 1}$, where *M* and *N* equal 420 and 12, respectively. Here, the first step involves the creation of a model’s output constant (initial) value [${\hat{f}}_{\left(0\right)}$] using Equation (1). In the following step, the scores (${\hat{g}}_{m}$) and canvases (${\hat{h}}_{m}$) are evaluated by employing Equations (2) and (3), respectively. This step involves all nodes (m=1 − M) having weak responses. As a result, the multi-objective condition [Equation (4)] can be solved using the training set $\left\{x_{i},-({\hat{g}}_{m}(x_{i}))/({\hat{h}}_{m}(x_{i}))\right\}$, as a basis for fitting the base learner (tree). The model is then iteratively enhanced in accordance with Equation (5), following a process of optimization. The final results are then evaluated using Equation (6), following the calibration of the model. In Equations (1)–(6), $L\left[y,\;f(x)\right]$ is a loss function that behaves differently depending on differentiability, and α is the learner’s rate of progress. It is important to note that, as part of the XG Boost single-tree analysis, $L\left[y,f(x)\right]$ is continually evaluated during the modeling process of each node to determine the node that will result in the highest gain over time. When features in ${\hat{f}}_{m}\left(x\right)$ are split into subsets, it is possible to create an additional regression tree. The accuracy of the model is calculated after adding up each predictor’s score to determine the total score for the model.

(1) $${\hat{f}}_{\left(0\right)}\left(x\right)=\arg_{\theta }min\sum_{i=1}^{N}L\left(y_{i},\theta \right)$$

(2) $${\hat{g}}_{m}\left(x_{i}\right)={\left[\frac{\partial L\langle y_{i},f(x_{i})\rangle \;\;}{\partial f\left(x_{i}\right)}\right]}_{f\left(x\right)={\hat{f}}_{\left(m-1\right)}\left(x\right)}$$

(3) $${\hat{h}}_{m}\left(x_{i}\right)={\left[\frac{\partial L\langle y_{i},f(x_{i})\rangle \;\;}{\partial f{\left(x_{i}\right)}^{2}}\right]}_{f\left(x\right)={\hat{f}}_{\left(m-1\right)}\left(x\right)}$$

(4) $$\begin{array}{c} {\hat{\phi }}_{m}=\mathrm{ar}g_{\phi \in \Phi }min{\sum_{i=1}^{N}\frac{1}{2}{\hat{h}}_{m}\left(x_{i}\right)\left[-\frac{{\hat{g}}_{m}\left(x_{i}\right)}{{\hat{h}}_{m}\left(x_{i}\right)}-\phi \left(x_{i}\right)\right]}^{2}\\ {\hat{f}}_{m\;}(x)=\alpha {\hat{\phi }}_{m}(x) \end{array}$$

(5) $${\hat{f}}_{m\;}\left(x\right)={\hat{f}}_{\left(m-1\right)}\left(x\right)+{\hat{f}}_{m\;}(x)$$

(6) $$\hat{f}\left(x\right)={\hat{f}}_{M}\left(x\right)=\sum_{m=0}^{M}{\hat{f}}_{m\;}\left(x\right)$$

### 2.3. Indicators of Prediction Performance

As a means of testing the accuracy of the developed models with respect to the test observations of the characteristic CS of SFRC, four performance metrics (Equations (7)–(10)) were calculated in this study. In these formulas, $a_{i}$, $\hat{a_{i}}$, and ${\bar{a}}_{i}$ are the tested, calculated, and mean of tested CS of SFRC’s, respectively.

(7) $$R^{2}=1-\frac{\sum_{i=1}^{n}{\left(a_{i}-\hat{a_{i}}\right)}^{2}}{\sum_{i=1}^{n}{\hat{a_{i}}}^{2}}$$

(8) $$RMSE=\sqrt{\frac{\sum_{i=1}^{n}{\left(a_{i}-\hat{a_{i}}\right)}^{2}}{n}}$$

(9) $$MAPE=\frac{100}{n}\sum_{i=1}^{n}\frac{\left|a_{i}-\hat{a_{i}}\right|}{\left|\hat{a_{i}}\right|}$$

(10) $$MSE=\frac{1}{n}\sum_{i=1}^{n}{\left(a_{i}-\hat{a_{i}}\right)}^{2}$$

## 3. Data Collection, Characteristics, and Handling

### 3.1. Data Compilation

In this study, the primary focus was on the 28d CS of SFRC with three types of mono-fibrous systems (crimped, hooked, and mil-cut) as a response to the SFRC ingredients and their characteristics. In total, the unprocessed population of the study consists of 422 datasets collected from 43 independent reports that were published between the period of 1994–2021. The careful selection of these reports was guided by a comprehensive evaluation of accessibility, data richness, and alignment with research objectives in order to ensure the robustness and relevance of the datasets. As listed in Table 1 and Table 2, the variables in the study are coded according to the database’s inputs (*X*) and output (*y*). Moreover, Table 3 provides a summary of the collected datasets that were used in the study.

The datasets for the current study were derived from the comprehensive database compiled by Wang et al. [8], which served as a basis for the present study. Their study focused on the mechanical characteristics of normal- and high-strength SFRC mixtures that only contained Portland cement (type I), natural aggregate, and a single type of steel fiber. A further aspect of ensuring the integrity of the compressive data has been achieved by considering the test specimen cylindrical with a diameter of 150 and a height of 300 mm. As a result, the conversion factors in Table 4 were applied to ensure consistency in the test results.

### 3.2. Data Wrangling and Statistical Analysis

#### 3.2.1. Treatment of Outliers

Statistically significant outliers are data points that deviate from the norm, which indicates there could be an anomaly in the data [100]. It is common practice in regression analysis to deal with outliers as the first factor, which can significantly impact the outcome [101]. Several factors can contribute to detecting an outlier in a given data set; these include errors in measurement, mistakes made in capturing data, and signals detected in newly acquired data. In statistical models and analyses, outliers pose a challenge, particularly when the data involved in the analysis are excessive [102,103]. However, outliers present an exciting opportunity for exploring new possibilities. It is possible to identify outliers using various methods based on the type of data being analyzed and the type of outlier being sought. Furthermore, these methods can detect emerging phenomena or anomalous behavior. Some methods, such as Chauvenet’s criteria and Grubb’s test, are available for identifying outliers that use averages and standard deviations and assume a normal data distribution [53].

**Figure 2:**
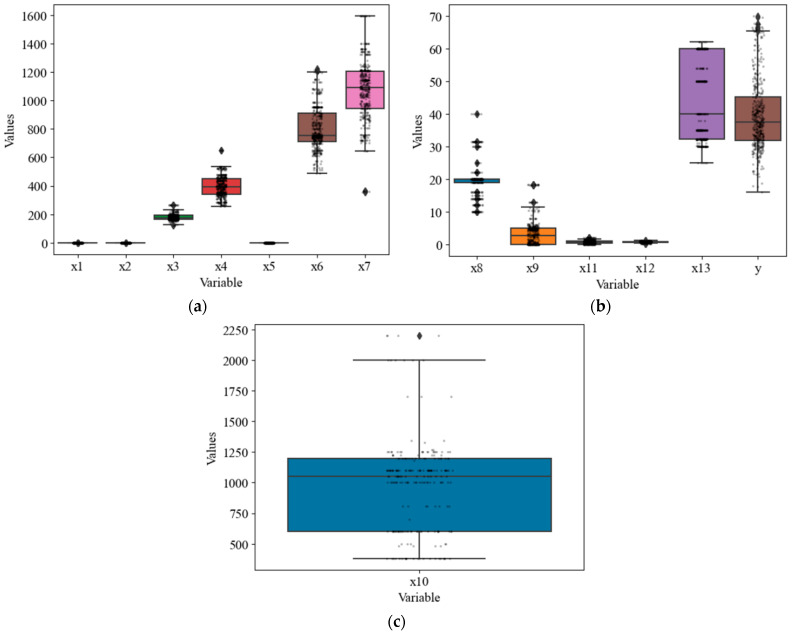
Boxplots of the model variables: (**a**) *X*1–*X*7, (**b**) *X*8, *X*9, *X*11–*X*13, *y*, and (**c**) *X*10.

The variables included in the study were analyzed using descriptive statistics according to the method described in [104] to identify any outliers. Here, Grubb’s test was used during preprocessing to detect outliers, errors, and even distributions in the data by checking them for outliers, errors, and odd distributions. To achieve this objective, we employed the p-test for hypothesis testing, employing a significance level of 5%. The null hypothesis posits that all data values originate from the same normal population, while the alternative hypothesis contends that the largest or smallest data value is an outlier It is noteworthy that further critical analysis of the data is an essential measure for determining the weaknesses of the approach to enhance its effectiveness. This analysis has been carried out via bivariate boxplots, confirming the datasets’ regularity. An evaluation of the rationality of the datasets has been conducted using bivariate boxplots (Figure 2), which indicated the rationality of the datasets for further regression analysis. This figure provides an informative summary of the distribution characteristics (e.g., median, interquartile range, outlier, and skewness) of model variables, making them valuable tools for data exploration.

#### 3.2.2. Data Descriptive Statistics and Visualization

**Figure 3:**
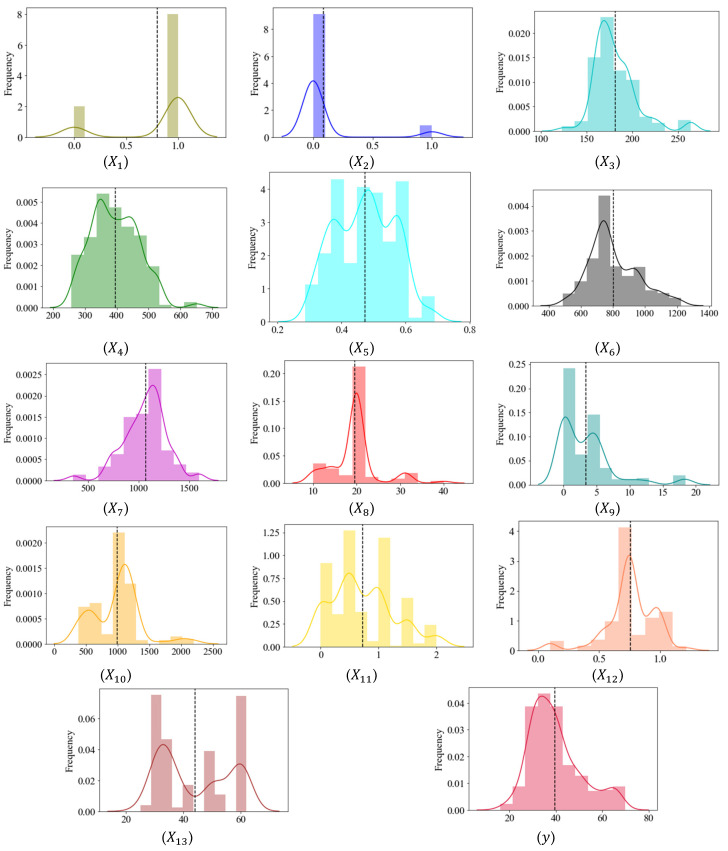
A graphic depiction of the model’s variables.

After cleaning up the database, two outliers were removed ((i) y=88  MPa, and (ii) $x_{7}=40\;$ mm), thus leaving 420 observations available for developing the AI model. A summary of the statistical information obtained from the refined database after removing outliers is presented in Table 5, while Figure 3 displays their graphical visualization. The figure demonstrates that the distribution of the majority of these variables is well-suited for applications in machine learning.

## 4. Results and Discussion

### 4.1. Features and Label Relations

**Figure 4:**
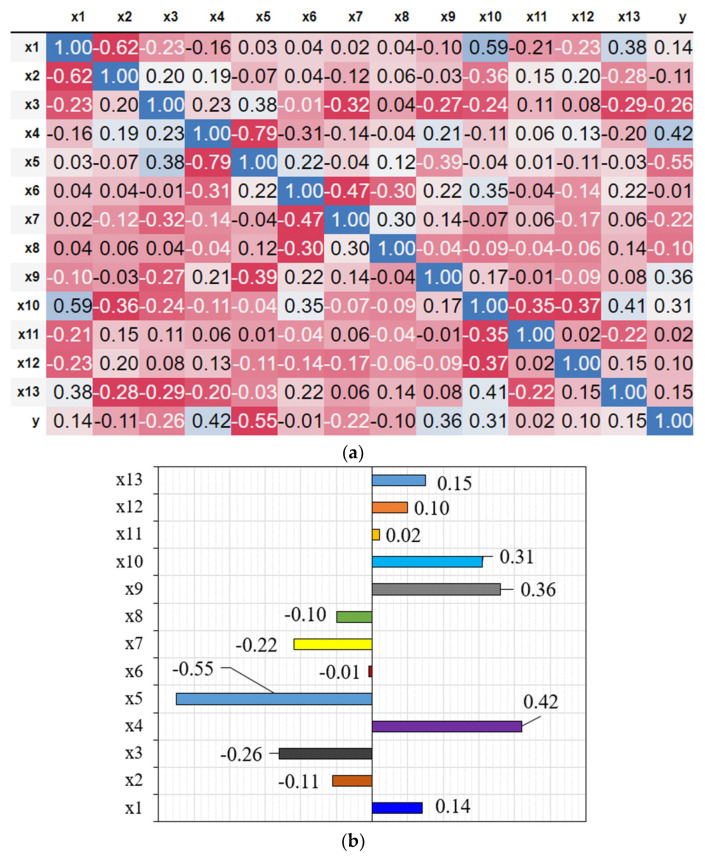
(**a**) Pearson’s encoded matrix, and (**b**) linear correlations between features and label.

In the current study, a Pearson correlation constant ($r_{xy},$ Equation (11)) was calculated during data preprocessing to assess the linear correlation between the model variables. A constant with a value between −1 and 1 is always present in this equation [105]. In this equation, n is the number of records, $(x_{i}$, $y_{i}$) is the number i feature–label set having an average value of $\bar{x},\bar{\;y}$. In a linear relationship between two random variables, the constant represents the average degree of variability of the linear relationship. The resulting correlation constant coefficients for the features–label relations are presented in Figure 4. As shown in the figure, cement, HRWR contents, and fiber tensile strength (i.e., $X_{4}$, $X_{8}$, and $X_{9}$) had the largest positive impact on SFRC CS. The results obtained here are consistent with those of Ayan et al. [106]. The study demonstrated that the type and quantity of binder, along with the volume fraction of steel fiber, exerted the most pronounced influence on the compressive strength of SFRC. In contrast, increasing the proportions of water–binder ratio, water, and coarse aggregates (i.e., $X_{5}$, $X_{3}$, and $X_{6}$) would likely reduce CS. This finding can be explained by poor microstructural properties and packing densities resulting from increased water–binder ratio and coarse aggregate contents [107]. Furthermore, a comprehensive examination of the data depicted in Figure 4 explains that variables *X*_6_ (fine aggregate content) and *X*_11_ (diameter of fiber) will likely exert minimal influence on the CS of SFRC. Given their negligible impact, these specific variables have been thoughtfully excluded from subsequent analyses and modeling.

(11) $$r_{xy}=\frac{\sum_{i=1}^{n}\left(x_{i}-\bar{x}\right)\left(y_{i}-\bar{y}\right)}{\sqrt{\sum_{i=1}^{n}\left(x_{i}-\bar{x}\right)}\sqrt{\sum_{i=1}^{n}\left(y_{i}-\bar{y}\right)}}$$

### 4.2. Development and Performance of the Initial Model

The default hyperparameters (Table 6) were used as a starting point for the development of the model (Model-0). It is worth noting that only the fine-tuned hyperparameters are included in this table. The prediction performance measures for this benchmark model are listed in Table 6. The initial model’s performance indicators for the test data were lower than those for the training data. Based on this finding, it appears that the initial model was prone to overfitting. Therefore, a multi-objective optimization process was used to fine-tune the default hyperparameters to maximize the model’s performance.

### 4.3. Fine-Tuned Model

**Figure 5:**
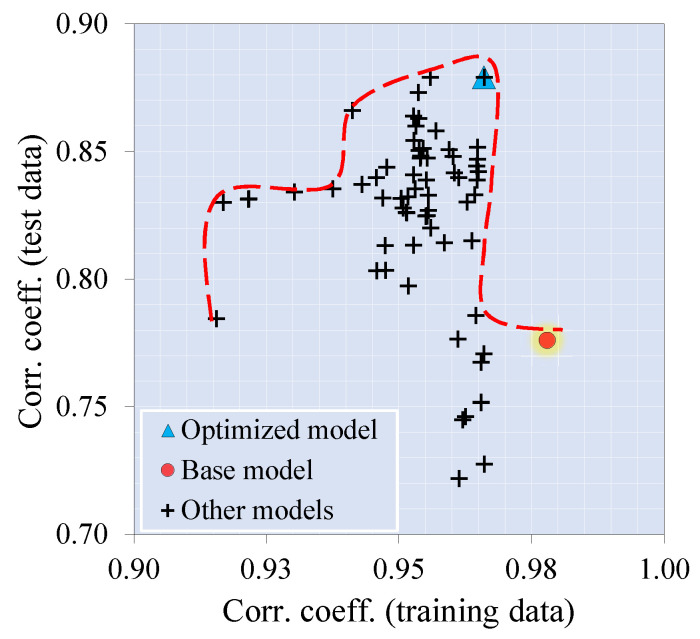
Pareto frontier-based model’s multi-objective optimization.

**Figure 6:**
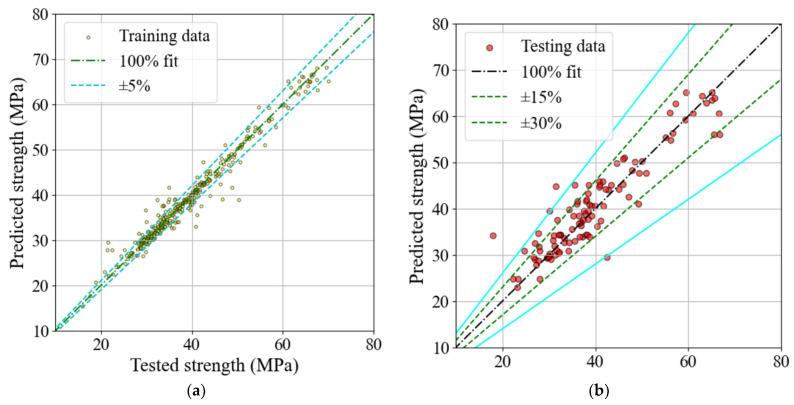
Predicted vs. target results for the: (**a**) training and (**b**) testing data.

In the present study, the hyperparameters (Table 6) most affecting the model’s performance were optimized by trial and error to achieve the best accuracy. In the pursuit of refining the model’s default hyperparameters, we adopted a multi-objective optimization strategy that combines aspects of both random search and grid search. At each iteration of the approach, we methodically documented the model’s performance, facilitating the identification of an optimal configuration that effectively balances diverse performance objectives, encompassing the $R^{2}$ scores for both training and testing data. A favorable result was achieved using a multitarget optimization technique based on Pareto’s [108] frontier approach. Figure 5 depicts the results of this multi-objective optimization process. The optimized model exhibited adequate prediction performance with scores of 0.966 and 0.879 (Table 7) for training and testing data. As presented in Figure 6, the model-target results were close to the ±95% and ±85% accuracy ranges for both training and testing results, respectively. Additionally, the error of the predictions by the constructed model rarely exceeds ±30%. The model seems to be able to make accurate predictions for the database used during the modeling process. The next stage of the study involved a narrowly focused experimental campaign to verify the accuracy of the proposed ML model. The following section provides details of the experimental programs.

### 4.4. Experimental Verification

#### 4.4.1. Materials

**Figure 7:**
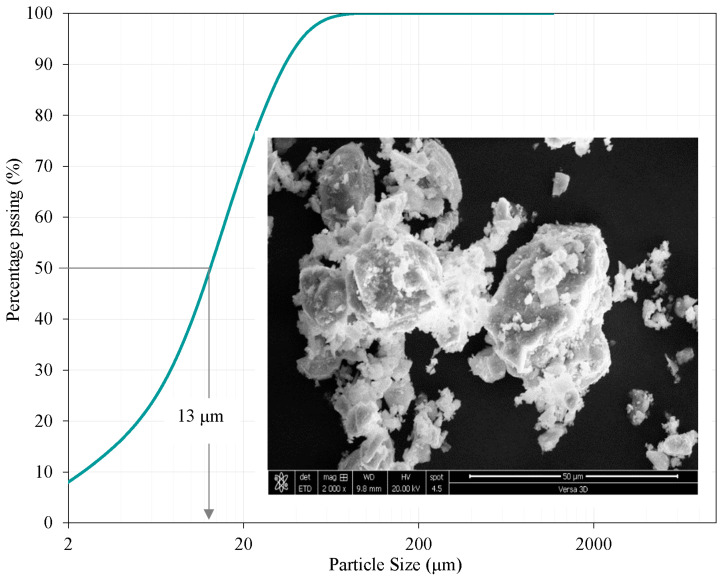
SEM analysis and particle-size curves for the used OPC.

In this investigation, we utilized ordinary Portland cement (OPC) type I, in accordance with ASTM C 150 [109], to formulate the SF-HSC, procured from a local manufacturing facility. The estimated median particle size of this cement is 13 microns. A comprehensive analysis of the physicochemical attributes of the OPC employed is detailed in Table 8, while Figure 7 depicts the grain size distribution. Additionally, Figure 7 presents a scanning electron microscopy (SEM) image of the OPC, revealing distinctive features such as polyangular shapes, an asymmetrical distribution, and particle sizes ranging from 1 to 20 µm.

The targeted workability for the concrete blends was successfully attained through the utilization of a modified polycarboxylic ether polymer, recognized as a high-range water-reducing agent (HRWR) and commercially known as MasterGlenium 51. This HRWR comprises 36% dry powder and possesses a relative density of 1.1, as specified by the manufacturer. To determine the appropriate quantity of HRWR to incorporate into the concrete mix, we divided the dry extract (D.E.) by the weight of the cement. Optimizing this ratio has resulted in achieving the optimal workability for this specific mix.

**Figure 8:**
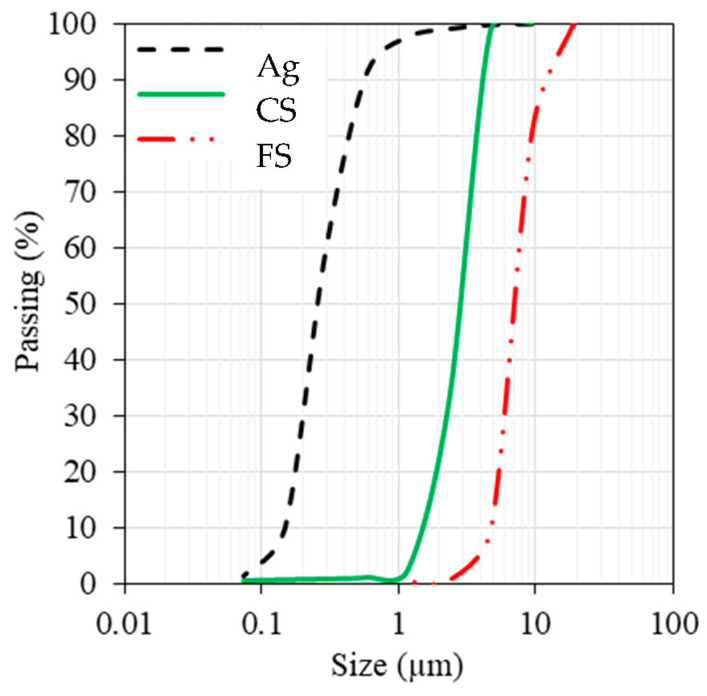
Particle-size curves for various aggregates.

Furthermore, all concrete blends were crafted with coarse aggregates (Ag) featuring a maximum aggregate size of 10 mm. The particle-size curves of the aggregates employed in this study are depicted in Figure 8.

The production of SFRC involved the incorporation of three distinct hook-ended steel fibers, each varying in both length and diameter. The steel fibers utilized encompassed a range of dimensions. A comprehensive overview of the physicomechanical characteristics of the employed steel fibers is provided in Table 9.

This investigation encompassed the formulation and assessment of 20 concrete blends, integrating three distinct water-cement ratios (0.25, 0.35, and 0.45), three varieties of fibers (as detailed in Table 9), and four levels of fiber dosage (0.0, 0.5, 1.0, and 1.5). The proportions of these mixes, along with the quantity of steel fibers incorporated in each, are outlined in Table 10. In this context, the labels U, H, and N signify concrete compositions featuring water–cement ratios of 0.25, 0.35, and 0.45, respectively. For instance, the designation “U-F1-0.5” indicates a mix prepared with a water–cement ratio of 0.25, utilizing steel fiber type “F1” at a dosage of 0.5 percent (volume). An important characteristic of the control blends is that the slump, serving as a measure of workability, was set at 100 ± 25 mm and assessed in accordance with ASTM C143 standards [110].

#### 4.4.2. Methods

##### Mixing, Casting, and Curing

Execution of this investigation involved blending various aggregates in a standard concrete mixer for several minutes, accompanied by the simultaneous introduction of absorption water. Subsequently, cement was dry-mixed for a brief duration. The high-range water-reducing (HRWR) agent was blended with water for two minutes, then re-mixed with the aggregates for three minutes, followed by an additional three minutes without mixing before the final two-minute blending phase. The resulting concrete mixture was poured into distinct molds, aligning with specimen size requirements, and the mixer was subsequently turned off. In the case of steel-fiber-reinforced concrete (SFRC) mixes, fibers were incorporated into the concrete mixture after a thorough initial mixing of five minutes to ensure optimal dispersion within the mixture.

Rigid plastic molds were utilized to cast a series of concrete cylinder specimens measuring 100 (dia.) × 200 (ht.) mm, assessing the compressive strength (CS) of the concrete. To maintain a conducive moisture environment, plastic sheets covered the specimens post-removal of excess material from the mold’s surface. Specimens were demolded after 24 h, and subjected to curing at 22 ± 2 °C with a relative humidity of 100%. The test specimens remained in this condition until the testing phase. Each type of test and mix underwent the casting of three specimens. For CS specimens, tests were conducted at both seven and 28 days. The study’s outcomes were then calculated, and average strength results for the three specimens were presented.

##### Method of Testing

**Figure 9:**
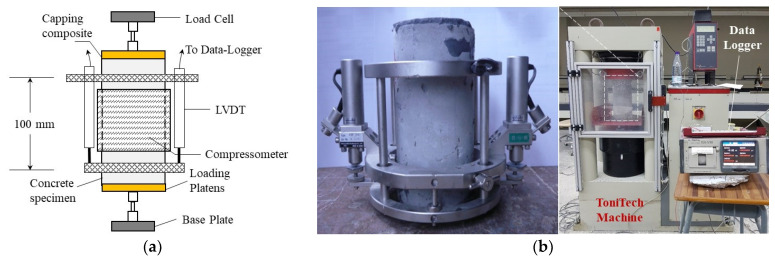
The uniaxial compression test: (**a**) representatives chart and (**b**) testing system.

Before subjecting the cylindrical specimens to the uniaxial compression test, a sulfur mortar coating is applied to ensure an even distribution of load across the top and bottom surfaces. This study assessed the compressive strengths (CSs) of cement-based materials at 7 and 28 days, adhering to ASTM C39 [111] specifications. Employing a ToniTech universal testing machine with a 3000 kN load capacity (depicted in Figure 9), the tests were conducted. The specimens were affixed with two linear variable displacement transducers (LVDTs) and a compressometer ring at a height of approximately 100 mm, corresponding to the center of the samples, to measure in-plane and transverse strains. Under displacement-controlled conditions, with a rate of 2.5 × 10^−3^ mm/s, the tests were executed. Each compressed test involved two or three duplicate samples, and the mean result was reported to ensure the reliability of the findings.

#### 4.4.3. Test and Model Results

**Figure 10:**
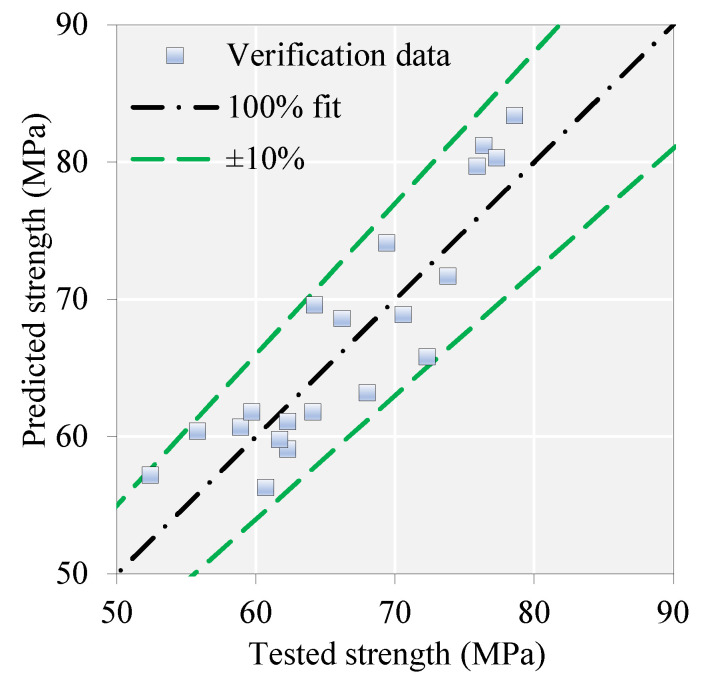
Predicted vs. target results for experimental verification data.

The observed and calculated CS of the studied SFRC mixes are listed in Table 10. The developed ML model yielded reasonable predictions. The mean and COV of the tested-to-predicted results were about 0.99 and 9%, respectively. A demonstration of this superior predictive capability is shown in Figure 10. In this figure, the predicted and tested data points show a low error rate of less than 10% in most cases.

## 5. Model Implementation

### 5.1. Feature Ranking

**Figure 11:**
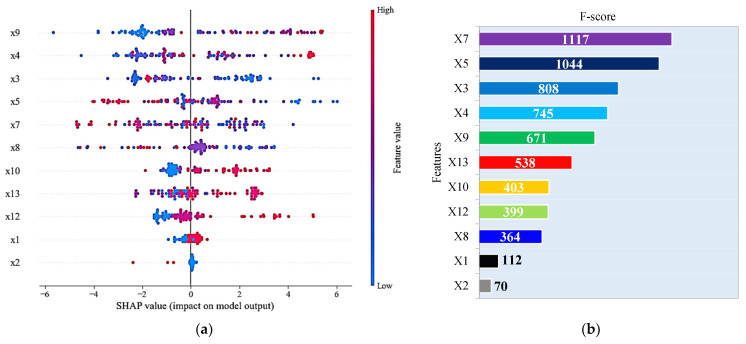
Variable significance by: (**a**) SHAP value, and (**b**) Gini index methods.

The analysis of influential variables in predicting the CS of SFRC plays a pivotal role in optimizing concrete mix designs for enhanced performance and durability. In this study, we employed two distinct approaches (Gini index [112] and Shapley additive explanations (SHAP)), to unveil the key factors that impact the CS of SFRC. These approaches provide valuable insights into the relative importance of different variables, shedding light on the interplay between various components within the concrete mixture. The practice of analyzing features based on Gini coefficients has proven to be more effective in detecting the significance of features with unique values [113]. The results of this analysis are shown in Figure 11.

The SHAP approach, a novel and robust tool for interpreting machine learning models, identified *X*_9_ (high-range water-reducer content), *X*_4_ (cement content), *X*_5_ (water–binder ratio), *X*_7_ (gravel content), and *X*_8_ as the most influential variables in predicting CS. The Gini index, another well-established technique for evaluating variable importance, identified a slightly different set of influential factors, namely *X*_7_, *X*_5_, *X*_3_, *X*_4_, *X*_9_, and *X*_13_. This divergence highlights the complementary nature of these two methods, as they provide unique perspectives on the significance of each variable. However, it is particularly noteworthy that both approaches unequivocally agree on four primary parameters significantly influencing the CS of SFRC: *X*_9_, *X*_4_, *X*_5_, and *X*_7_. Here, *X*_9_, representing the high-range water-reducer content, plays a crucial role in optimizing the workability and strength of the concrete mixture. Additionally, the cement content (*X*_4_) stands as a pivotal factor, as confirmed by Figure 11a, which visually depicts how higher cement content positively correlates with increased strength, while lower content results in reduced strength. The water–binder ratio (*X*_5_) and gravel content (*X*_7_) are also integral components, with their appropriate proportions contributing to the desired CS in SFRC.

It is noteworthy that the consistency in the rankings of other variables, such as coarse aggregate content (*X*_6_) and fiber type (*X*_1_ and *X*_2_), between the two methods, adds further credibility to our findings. These results not only underscore the robustness of our analysis but also provide valuable insights for optimizing SFRC mix designs. Importantly, these findings align with the results reported in Section 4.1 of this study, where the Pearson correlation analysis also highlighted the significance of *X*_5_, *X*_4_, and *X*_9_ in predicting the CS of SFRC. This collective evidence strengthens the confidence in the identified key parameters and their impact on the compressive strength of SFRC, offering a valuable foundation for future concrete mix design optimization efforts.

### 5.2. Partial Dependence Plots

**Figure 12:**
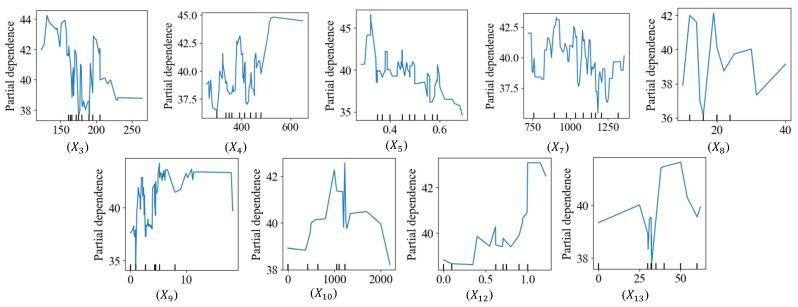
Compressive strength of SFRC partial dependence plots.

This study conducted a partial dependence analysis for each independent variable employed in the ML model. Figure 9 shows the PDPs of the CS of SFRC in response to different predictors, except the dummy ones. The figure suggests the optimum water content ($X_{3}$) and HRWR ($X_{8}$) are in the range of 100–150 kg/m^3^ and more than 10–20 kg/m^3^ to maximize the CS, and strength notably decreases as the content increases. Further, the strength will likely increase as the cement content ($X_{4}$) increases. Additionally, the ideal content for coarse aggregate ($X_{7}$) content is perhaps 900–1100 kg/m^3^. Moreover, the results in the figure suggest that the best fibrous combination has a tensile strength ($X_{10}$) of about 1000 MPa, dosage ($X_{12}$) of around 1.0%, and length ($X_{13}$) of 40–50 mm. As expected, Figure 12 also illustrates that the CS decreases as the water–binder ratio ($X_{5}$) increases. 

### 5.3. Graphical User Interface Development

**Figure 13:**
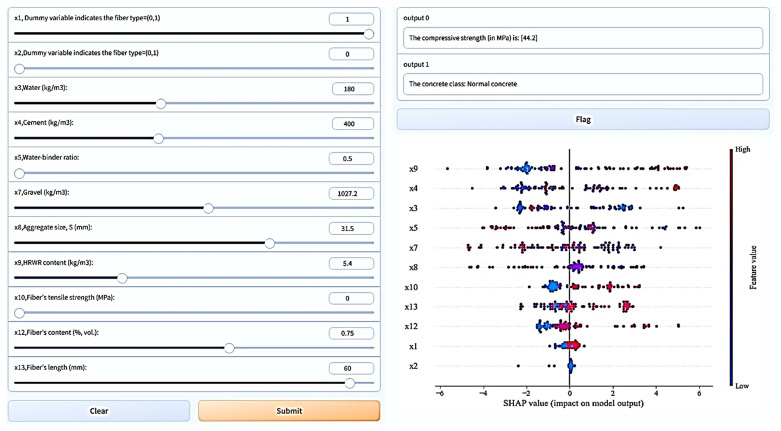
A GUI for predicting the compressive strength of SFRC using the XG Boost model.

In this study, we provide an intuitive graphical user interface (GUI) for interacting with the developed XG Boost model. Python and Gradio [114] have been used to implement sliding control systems that allow input values to be limited to minimums and maximums (Table 5). Figure 13 shows three main components: input features with slider controls, output results, and SHAP-based explanations. The model produces the SFRC’s strength and the concrete class (“normal strength” if it has a strength lower than 60 MPa, otherwise “high-strength concrete”).

## 6. Conclusions, Implications, and Future Research

This study involved the compilation and refinement of an extensive database, comprising 420 entries sourced from 43 scholarly publications. We conducted experimental analyses on 20 different SFRC mixtures to assess the predictive accuracy of the constructed model. Furthermore, we employed PDPs to elucidate the relationships between the model’s input variables and its outcomes. The significance of these input variables within the model was also explored. To enhance the model’s usability, we developed a user-friendly graphical interface. It is worth noting that the research specifically focused on three distinct fiber types: crimped, hooked, and mil-cut. Therefore, the findings may not be directly applicable to SFRC with alternative fiber types or mixtures. Additionally, while the model consistently performed well with experimental data, its effectiveness may vary under different conditions or when using different raw materials. Regarding the research findings:

1. The analysis, including Pearson correlations, Gini indices, and SHAP analyses, highlighted that the most significant factors influencing the CS of SFRC were the cement and HRWR contents, as well as the fiber tensile strength and water–binder ratio. Notably, increasing the proportion of water and coarse aggregates is likely to reduce the compressive strength of the concrete.
2. We utilized the Pareto frontier multi-criterion method to develop an optimized version of the standard XG Boost model. Based on training and testing datasets, the optimized model demonstrated satisfactory predictive performance, achieving scores of 0.97 and 0.88, respectively.
3. The developed ML model consistently exhibited superior predictive capability when tested against independent experimental data conducted by the authors, with average and COV values of the tested-predicted results at 0.99 and 6%, respectively.
4. Through the application of PDPs, we determined that the optimal water and HRWR contents for achieving maximum CS are in the range of 100–150 kg/m^3^ and 10–20 kg/m^3^, respectively. Similarly, for coarse aggregates, ideal contents fall in the ranges of 900–1100 kg/m^3^. Additionally, the most effective fibrous combination exhibited a tensile strength of 1000 MPa, a diameter length of 40–50 mm, and a dosage of about 1.0%.

The adoption of ML techniques, particularly the XG boosting methodology, offers the construction and civil engineering sectors an enhanced predictive toolset for determining the CS of SFRC. This research not only elucidates optimal SFRC formulations, pinpointing effective fiber combinations but also facilitates the development of concrete with superior strength and durability. In future investigations, it would be valuable to compare the predictive competence of the current numerical model against existing empirical and analytical frameworks. Additionally, the inclusion of data on ultra-high-performance concrete could enhance the model’s universality. Addressing the size effect might benefit from the incorporation of a conversion factor as an input variable for various types of test samples, effectively contributing to handling this aspect of the study. While our present research offers a robust database, there is a compelling case for expanding this repository by incorporating newer studies and a wider array of SFRC mix variations, ensuring it remains at the forefront of technological progress. Despite the current study’s reliance on the XG boosting technique, exploring alternative ML schemes (e.g., neural networks or stacked ensemble algorithms) may reveal novel perspectives and enhance predictive accuracy. Beyond immediate CS predictions, there is a growing need to investigate the enduring resilience of SFRC in diverse scenarios. In an era emphasizing ecological responsibility, future research should critically assess the environmental implications of various SFRC formulations, including aspects such as lifecycle assessments, carbon emissions, and potential for recycling. Furthermore, forthcoming research endeavors could explore the impact of fiber orientation on the post-cracking behavior of SFRC under compressive loading.

## Figures and Tables

**Table 1:** The coding system for the dummy variables.

Fiber Type	Hooked	Mil-Cut	Crimped
$X_{1}$	1	0	0
$X_{2}$	0	1	0

**Table 2:** The coding system for the non-dummy variables.

Variable	Unit	Description	Variable	Unit	Description
$X_{3}$	kg/m^3^	Water content	$X_{9}$	kg/m^3^	HRWR content
$X_{4}$		Cement content	$X_{10}$	MPa	Tensile strength of the fiber
$X_{5}$	––	Water–binder ratio	$X_{11}$	mm	Diameter of the fiber
$X_{6}$	kg/m^3^	Fine aggregate content	$X_{12}$	%, vol.	Dosage of the fiber
$X_{7}$		Coarse aggregate content	$X_{13}$	mm	Length of the fiber
$X_{8}$	mm	Maximum aggregate size	y	MPa	28d CS of the SFRC

Note. HRWR: high-range water reduction superplasticizer.

**Table 3:** Summary of the collected CS database of SFRC.

Ref.	Data- Sets	$X_{1}$	$X_{2}$	$X_{3}$	$X_{4}$	$X_{5}$	$X_{6}$	$X_{7}$	$X_{8}$	$X_{9}$	$X_{10}$	$X_{11}$	$X_{12}$	$X_{13}$	y
[58]	17	1	0	180–192.5	280–400	0.5|0.6|0.7	713.8–762.6	1027.2–1097.4	31.5	3.4–6.6	––	0.19–0.64	0.55–0.9	30–60	29.7–46.7
[59]	12	1	0	162–177	354–450	0.4|0.5	893–920	858–887	19	2.3–6.5	1050	0–1	0.75	60	31.5–53.3
[60]	4	1	0	181.2	453	0.4	624	1242	22	1	1100	0–0.8	0.75	60	21.7–25.3
[61]	5	1	0	230.2	338	0.7	1049.2	760	10	0.7	––	0–1.25	0.75	60	25.6–28.1
[62]	40	1	0	175	350	0.5	952.2	1321.2–1398.8	20	4.5	1100–1250	0–1.5	0–1.5	50–60	17.9–40.8
[63]	14	1	0	178–179	325–396	0.4|0.6	842–891	913–965	19	0.9–1.4	1050–2000	0–1	0.71–0.75	60	47.2–61.1
[64]	2	1	0	175.6	351	0.5	914.8	994.7	22	2.7	––	0.26–0.45	1	50	33.3–33.4
[65]	5	1	0	205–398	311–458	0.6|0.7	408–665	1156–1407	20	3.1–7.3	1700	0–1	0.4	25	31.2–88.0
[66]	2	1	0	169.8–177.6	283–296	0.6	705–838	968–1071	10	2.8–3.3	––	1–1.5	0.62	30	23.3–30.0
[67]	7	1	0	220	440	0.5	1193–1225	356–366	10	3–4	––	0–1.5	0.75	25–31	35.1–43.8
[68]	8	1	0	191.2–191.6	324	0.6	1052.8–1064.2	750.0–758.3	22	3.2	1200	0.19–0.76	0.5–0.75	30–60	34.0–48.0
[69]	2	1	0	185	410	0.5	915	866	20	2–4	1100	0.5–1.0	1	50	50.0–52.0
[70]	10	0|1	0	150–168	341–429	0.3|0.4|0.5	800	975	14	2.8–11	––	0.51	1	54–60	40.9–67.6
[71]	18	1	0	123–167	325–439	0.6	741–885	846–998	14	0.5–11.4	––	0.48–0.75	1	54–60	38.2–69.6
[41]	6	1	0	195	342	0.3|0.5|0.6	701	1143	20	10.3	––	0–1.5	0.75	30	24.6–30.6
[72]	23	1	0	164–185	308.3–529.1	0.3|0.6	639.3–763.9	928.6–1232.7	20	0–5.8	600–1000	0-2	0.55–0.75	35	26.7–65.4
[73]	32	1	0	172–195	336–521	0.6	652–750	1080–1145	10–40	0–5.2	1100	0-1	0.75	30–60	25.8–67.4
[74]	21	0|1	0	166	286	0.4	739	1170–1259	20	0–0.6	600	0–2	0.75	30–40	28.0–33.0
[40]	5	0	0	165	367	0.3|0.4|0.5	702–765	1053–1146	16	2.2	380	0–2	0.5	30	26.8–31.0
[75]	12	1	0	195–200	415–651	0.4|0.7	527–610	1022–1182	20	0	1000	0–1.5	0.75	32	30.9–58.5
[76]	6	1	0	185	268–524	0.4|0.5|0.6	488–725	1056–1185	31.5	0	1000	0–0.63	1	50	16.0–34.4
[77]	22	0|1	0|1	172–196	321–475	0.4|0.5	622.8–886.0	960–1171	20	0–4.5	––	0–2	0.75–0.94	32.3–62	26.6–43.6
[78]	6	1	0	156–165	312–381	0.6	817–1200	700–1214	20	2.2–5.3	1000	0.38–0.77	1	50	63.9–67.5
[79]	13	0|1	0	264	480	0.4	716.5–769.1	895.1–989.5	20	0	380–500	0–2	0.9–1.2	30.2–32.3	27.7–34.3
[80]	4	1	0	175	461	0.4	512	1252	10	0	600	0–1.5	1.0	35	28.5–31.4
[81]	2	1	0	160	400	0.4|0.6	750	1140	20	8	1700	0–0.5	0.5	30	42.7–45.1
[82]	2	1	0	230	535	0.5	556	1079	16	0	1345	0–1.0	0.6	30	36.5–41.1
[42]	2	0	0	165	300–471	0.4	690–760	1038–1140	20	1.8–2.8	380	1	0.5	30	23.8–39.0
[83]	18	0	1	195–228	361–475	0.4	630–875	715–1180	20	0	380	0–2	0.94	32.2	28.1–34.1
[84]	2	1	0	166	415	0.4	838.6	1024.9	20	0	1345	0–1	0.55	35	40.8–42.6
[85]	5	0	1	161	460	0.4	1150	1048.8	20	18.4	808.6	0–1	0.8	32	30.0–40.6
[86]	4	0	1	215.5	480	0.4	825	880	25	8	––	0–0.51	0.5	38	40.6–49.3
[87]	16	0|1	0|1	160–200	258–513	0.4|0.5|0.6	540–868	1012–1283	20	0	500–1325	0–0.7	0.55–1.15	32–50	27.0–47.0
[88]	5	1	0	172	400	0.4	730	1046–1100	15	0	––	0	0.8	32	27.7–37.0
[89]	10	0	0	161.0–167.7	453.3–460.0	0.4	699.2	1594.2	20	18.1–18.4	808.6	0–1.6	0.8	32–40	43.5–56.0
[90]	28	1	0	161.7	437	0.4	756	1210	20	1.3–6.1	––	0–1.5	0.7	35–50	31.8–50.3
[91]	3	1	0	168.1	410	0.4	1073	645	16	4.1–5.7	1100	0–1	0.8	50	32.1–38.7
[92]	3	1	0	167.7	390	0.4	1075	758	16	2.3–3.1	1100	0–1.45	0.8	50	36.9–40.5
[93]	9	1	0	198–205	440–460	0.4|0.5	924–985	721–846	12	11.1–12.8	1225	0.76	0.62	40	52.4–70.0
[94]	5	1	0	165	300	0.6	1128.8	806.3	16	2.5	1050–1100	0–0.5	0.55–0.75	35–60	17.6–29.2
[95]	6	1	0	190	380	0.6	1082.0	742.0	12	0.0–2.4	2200	0–0.63	0.35	30	21.6–43.92
[96]	3	1	0	169.1	412.4	0.4	927.8	890.7	12	0.0	1100	0–0.63	1	50	40.1–41.6
[97]	3	1	0	188.0	400.0	0.5	610.0	1132.0	10	3.3	1270	0–1	0.62	30	36.4–40.5
Tot.	422	0|1	0|1	123–398	258–651	0.3–0.7	408–1225	356.0–1594.2	10–40	0.0–18.4	380–2200	0–2	0.1–1.2	25–62	16–88

Note. In this table, $X_{5}$ is rounded to one decimal place.

**Table 4:** Data consistency conversion factors.

Specimen Type	Conversion Factor	Source	Note
Cubic	0.80	ACI 318 [98]	Below 60 MPa strength
Cubic	0.90	ACI 318 [98]	Above 60 MPa strength
Prismatic	0.96	Wu et al. [99]	––

**Table 5:** Descriptive statistics of the processed datasets.

Variable	Mean	StDev	Minimum	Q1	Median	Q3	Maximum
$X_{1}$	0.8012	0.3997	0.0000	1.0000	1.0000	1.0000	1.0000
$X_{2}$	0.0865	0.2814	0.0000	0.0000	0.0000	0.0000	1.0000
$X_{3}$	181.19	23.62	123.00	165.00	175.00	192.50	264.00
$X_{4}$	394.97	74.01	258.00	342.00	392.00	450.00	651.00
$X_{5}$	0.472	9.23	0.285	0.390	0.4800	0.550	0.690
$X_{6}$	804.05	152.58	488.00	713.00	756.00	911.00	1225.00
$X_{7}$	1067.6	210.6	356.0	945.0	1088.6	1206.3	1594.2
$X_{8}$	19.542	5.218	10.000	19.000	20.000	20.000	40.000
$X_{9}$	3.451	4.131	0.000	0.000	2.750	4.963	18.400
$X_{10}$	989.4	378.5	380.0	600.0	1050.0	1200.0	2200.0
$X_{11}$	0.7246	0.5377	0.0000	0.3840	0.5780	1.0000	2.0000
$X_{12}$	0.7553	0.1970	0.1000	0.7000	0.7500	0.9000	1.2150
$X_{13}$	44.015	12.178	25.000	32.300	40.000	60.000	62.000
y	39.677	11.034	16.000	31.800	37.418	45.358	70.000

**Table 6:** XG Boost hyperparameters for the initial and fine-tuned models.

Hyperparameter	Role	Range	Default Value (Model-0)	Optimized Value (Model-1)
max_depth	Tree maximum depth: modifying this parameter to higher values results in a more intricate model, increasing the risk of overfitting.	0–∞	6	52
n_estimators	This hyperparameter dictates the quantity of boosting iterations or trees incorporated within the ensemble.	1–∞	100	325
learning_rate	Step-size shrinkage employed during updates is intended to mitigate overfitting.	0–1	0	0.2
colsample_bytree	The subsample ratio of columns determines the proportion of features used when constructing each tree.	0–1	1	0.1
subsample	Subsample ratio of training instance (e.g., 0.5 indicates 50% of data used prior to growing trees).	>0–1	1	0.5
reg_alpha	L1 regularization: increasing its value makes the model more conservative.	––	0	0.01
reg_lambda	L2 regularization: increasing its value makes the model more conservative.	––	1	10
gamma	Regularization parameter for tree pruning that specifies the minimum loss reduction required to make a split.	0–∞	0	0.1

**Table 7:** Performance metrics of the initial and fine-tuned models.

Performance Indicator	Model-0		Model-1	
	Training Set	Testing Set	Training Set	Testing Set
MAPE	0.742	3.541	1.239	2.797
NMBE	2.576	28.413	4.008	16.997
RMSE	1.605	5.330	2.002	3.933
$R^{2}$	0.978	0.776	0.966	0.879

**Table 8:** The physicochemical properties of the used OPC.

Oxide Composition (%)							L.O.I. (%)	Specific Gravity	Fineness (m^2^/kg)
SiO_2_	Al_2_O_3_	Fe_2_O_3_	CaO	MgO	Na_2_Oeq	SO_3_			
20.20	5.49	4.12	65.43	0.71	0.26	2.61	1.38	3.14	373

**Table 9:** Properties of the used steel fibers.

Fiber		Length (mm)	Diameter (mm)	Aspect Ratio	Young’s Modulus (GPa)	Tensile Strength (MPa)
Shape	ID					
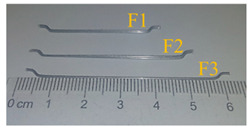	F1	40	0.62	65	210	1250
	F2	50	0.62	80		
	F3	60	0.75	80		

**Table 10:** Test features and response.

		Model’s Input Variables													Strength (MPa), y		$y_{1}/y_{2}$
No.	ID	$X_{1}$	$X_{2}$	$X_{3}$	$X_{4}$	$X_{5}$	$X_{6}$	$X_{7}$	$X_{8}$	$X_{9}$	$X_{10}$	$X_{11}$	$X_{12}$	$X_{13}$	$\mathrm{Test},\;y_{1}$	$\mathrm{Model},\;y_{2}$	
1	H-F1-0.5	1	0	157.5	450	0.35	716	1053	10	0.68	1250	0.62	0.5	65	69.4	74.1	0.937
2	N-F1-0.5	1	0	157.5	350	0.45	798	1078	10	0.42	1250	0.62	0.5	65	60.7	56.3	1.078
3	H-F1-1.0	1	0	157.5	450	0.35	716	1053	10	0.68	1250	0.62	1.0	65	72.3	65.8	1.099
4	N-F1-1.0	1	0	157.5	350	0.45	798	1078	10	0.42	1250	0.62	1.0	65	64.1	61.8	1.037
5	H-F1-1.5	1	0	157.5	450	0.35	716	1053	10	0.68	1250	0.62	1.5	65	75.9	79.7	0.952
6	N-F1-1.5	1	0	157.5	350	0.45	798	1078	10	0.42	1250	0.62	1.5	65	62.3	59.1	1.054
7	H-F2-0.5	1	0	157.5	450	0.35	716	1053	10	0.68	1250	0.62	0.5	80	73.8	71.7	1.029
8	N-F2-0.5	1	0	157.5	350	0.45	798	1078	10	0.42	1250	0.62	0.5	80	55.8	60.4	0.924
9	H-F2-1.0	1	0	157.5	450	0.35	716	1053	10	0.68	1250	0.62	1.0	80	76.4	81.2	0.941
10	N-F2-1.0	1	0	157.5	350	0.45	798	1078	10	0.42	1250	0.62	1.0	80	62.3	61.1	1.020
11	H-F2-1.5	1	0	157.5	450	0.35	716	1053	10	0.68	1250	0.62	1.5	80	77.3	80.3	0.963
12	N-F2-1.5	1	0	157.5	350	0.45	798	1078	10	0.42	1250	0.62	1.5	80	64.2	69.6	0.922
13	H-F3-0.5	1	0	157.5	450	0.35	716	1053	10	0.68	1250	0.75	0.5	80	68.0	63.2	1.076
14	N-F3-0.5	1	0	157.5	350	0.45	798	1078	10	0.42	1250	0.75	0.5	80	58.9	60.7	0.970
15	H-F3-1.0	1	0	157.5	450	0.35	716	1053	10	0.68	1250	0.75	1.0	80	70.6	68.9	1.025
16	N-F3-1.0	1	0	157.5	350	0.45	798	1078	10	0.42	1250	0.75	1.0	80	59.7	61.8	0.966
17	H-F3-1.5	1	0	157.5	450	0.35	716	1053	10	0.68	1250	0.75	1.5	80	78.6	83.4	0.942
18	N-F3-1.5	1	0	157.5	350	0.45	798	1078	10	0.42	1250	0.75	1.5	80	66.2	68.6	0.965
19	H-CTRL	1	0	157.5	450	0.35	716	1053	10	0.68	––	––	––	––	61.7	59.8	1.032
20	N-CTRL	1	0	157.5	350	0.45	798	1078	10	0.42	––	––	––	––	52.4	57.2	0.916
																μ	0.992
																σ	0.058
																CV	0.059

## Data Availability

Data are contained within the article.
